# Identification of Gait-Cycle Phases for Prosthesis Control

**DOI:** 10.3390/biomimetics6020022

**Published:** 2021-03-26

**Authors:** Raffaele Di Gregorio, Lucas Vocenas

**Affiliations:** LaMaViP, Department of Engineering, University of Ferrara, 44122 Ferrara, Italy; lucas.vocenas@edu.unife.it

**Keywords:** above-knee amputee, prosthesis, gait cycle, proprioception, lower-limb control

## Abstract

The major problem with transfemoral prostheses is their capacity to compensate for the loss of the knee joint. The identification of gait-cycle phases plays an important role in the control of these prostheses. Such control is completely up to the patient in passive prostheses or partly facilitated by the prosthesis in semiactive prostheses. In both cases, the patient recovers his/her walking ability through a suitable rehabilitation procedure that aims at recreating proprioception in the patient. Understanding proprioception passes through the identification of conditions and parameters that make the patient aware of lower-limb body segments’ postures, and the recognition of the current gait-cycle phase/period is the first step of this awareness. Here, a proposal is presented for the identification of the gait-cycle phases/periods under different walking conditions together with a control logic for a possible active/semiactive prosthesis. The proposal is based on the detection of different gait-cycle events as well as on different walking conditions through a load sensor, which is implemented by analyzing the variations in some gait parameters. The validation of the proposed method is done by using gait-cycle data present in the literature. The proposal assumes the prosthesis is equipped with an energy-storing foot without mobility.

## 1. Introduction

Human locomotion is an efficient biomechanical process. A healthy individual can travel long distances with low energy consumption. Despite the progress in prosthetic design, the replacement of lower-limb segments with a prosthesis affects the efficiency of this locomotion. The purpose of a lower-limb prosthesis is to minimize the impact of the amputation and make the patient somehow autonomous again. That is why prosthesis technology mainly tries to mimic the joint behavior of human lower limbs during walking.

The study of asymptomatic walking, therefore, presents the basis for thinking about the development of prosthetic components [1]. It seems indeed judicious to have maximum data on the movement, which one seeks to reproduce. Unfortunately, although walking seems relatively simple to healthy people, since it does not require any concentration, it is an extremely complex process to model, involving many mechanisms. The complexity of this modeling explains the large number of works dedicated to it (see [2,3] for References).

Numerous studies carried out over the years have laid the foundations for the various techniques of the current analysis. There are many different walking models, but none of them are completely satisfactory, and the research on optimal modeling is still in progress [2,3,4,5,6,7,8,9,10,11,12,13,14,15]. Each observation technique of human walking has limits, and many are the external factors that influence the measurements, which explains the disparity of results present in the literature [16].

**Figure 1:**
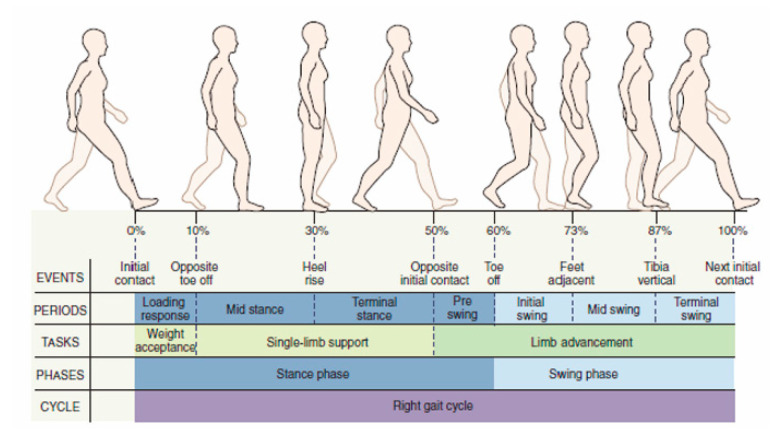
Gait-cycle phases (reproduced with permission from [19]).

Each person has a specific gait. However, a pattern common to all individuals is identifiable: the walking (or gait) cycle (Figure 1). Indeed, walking is substantially a repetitive activity; thus, its analysis refers to the motion cycle repeated during walking. Two successive impacts on the ground of the same heel delimit one cycle. The analysis of the gait cycle can consider only one (named “ipsilateral limb”) out of the two lower limbs, which is usually the right leg. These aspects are independent of individual characteristics, whereas stride, step length, step width, step angle, and cadence (see [17,18] for definitions) vary according to the physical characteristics of individuals and, for the same individual, according to his/her physiological conditions. There are two different phases during the gait cycle [17,18]: the stance phase and the swing phase (Figure 1). The stance phase, which corresponds to 60% of the cycle in the “normal” gait^1^, occurs when the foot of the ipsilateral limb is in contact with the ground. It begins with the “initial contact (IC)” event, which is when the heel touches the ground, and ends with the “toe-off (TO)” event, which is when the toe is lifted. During this phase, the body weight is transferred from the rear leg to the front leg. The swing phase, which corresponds to 40% of the cycle in the “normal” gait, occurs when the foot does not touch the ground and the leg oscillates. It begins with the TO event and ends with the next IC event.

If both the limbs are considered [18], the stance phase can be further divided into three periods: two “double-support” periods (i.e., periods in which both the feet touch the ground), one at the beginning (initial double support) and the other at the end (terminal double support), and a third, “single-limb support” period for the remaining part. The extension in the percentage of the cycle of these three periods depends on the walking speed. In addition, it is worth mentioning a finer repartition [19], which refers to seven events that make it possible to identify four periods in the stance phase and three periods in the swing phase (Figure 1).

The observation of the three-dimensional kinematics of the gait cycle can relate the joint angles of the knee and ankle to a particular percentage of gait-cycle completion [20]. Consequently, an attempt of obtaining the postures of lower-limb body segments by identifying the gait-cycle phases/periods may succeed. The same observation reveals that, during the gait cycle, the position of the center of mass oscillates both vertically and laterally [19].

The dynamic analysis of the gait cycle characterizes the force systems both externally and internally (i.e., in the joints) acting on the lower limbs. The dynamic equilibrium of the pedestrian can be stated by saying that the ground reaction forces (GRFs), which the ground applies to the feet, must equilibrate the force system consisting of the body weight and the inertia forces. Therefore, measuring the three GRF components (i.e., vertical force, anterior–posterior shear, and medial–lateral shear) provides relevant pieces of information on the gait cycle phase/period. The GRF is non-null only during the stance phase. During this phase [19,21,22], in the normal gait:

- The vertical force has two peaks of about 120% of the body weight (BW) that occur approximately at the end of the initial double-support period (i.e., 10% of gait-cycle completion (Figure 1)) and earlier than the beginning of the terminal double-support period (i.e., 45–50% of gait-cycle completion (Figure 1)) with a minimum of about 80% BW in the middle (i.e., 30% of gait-cycle completion (Figure 1));
- The anterior–posterior shear has two peaks with opposite signs of about 20% BW, which approximately occur when the vertical force has two peaks and vanish in the middle of the stance phase (i.e., 30% of gait-cycle completion (Figure 1));
- The medial–lateral shear is much smaller than the other two components. It has no sharp peaks, and it is comprised in the range [−5, +5]%BW

These results and GRF diagrams reported in the literature [1,17,18,19,21,22] make it possible to relate a signal obtained by measuring the GRF components to the kinematic data of the ankle and knee joints to recognize the current gait-cycle phase/period.

Active/semiactive lower-limb prostheses have been extensively studied in the last two decades [23,24,25,26,27,28,29,30,31,32,33], and up to 21 [32] or 26 [31] types of active prostheses, according to the adopted classification criterion, have been counted in recent reviews, among which 12 are for above-knee amputees. Three different types of control systems have been adopted for them: echo control, gait-mode control, and, recently, direct myoelectric control. Echo control aims at mimicking the motion of the healthy limb. It is applicable only to unilateral amputees and needs the introduction of sensors both on the healthy limb and on the prosthesis; it is not able to reproduce asymmetric walking. Gait-mode control tries to recognize the current gait-cycle phase/period by means of a number of sensors inserted into the prosthesis and adapts the prosthesis behavior to the current gait-cycle phase/period by using software based on artificial-intelligence algorithms. Direct myoelectric control interprets the signals coming from the contraction of the stump muscles of the patient to control the prosthesis behavior. It is a novel approach aiming at making the patient able to generate prosthesis commands with his/her brain. At the moment, the gait-mode control guarantees better performances [32], and it is implemented into commercial active/semiactive knee prostheses [33].

This paper presents a method for the detection of the gait-cycle events on different gait conditions through a load sensor and its use for controlling an active/semiactive prosthetic knee. The method is based on the analysis of the variations of a number of gait parameters. The experimental data on the asymptomatic gait reported in the literature are exploited to establish the control model. Only the behavior of a prosthetic knee equipped with an energy-storing foot without mobility is considered in this work.

Differently from other gait-mode prosthesis control, the proposed method allows devising a control logic based only on real-time GRF measurements and on deterministic computations. This approach reduces the prosthesis hardware and simplifies the control software, thus moving toward low-cost prescriptions and increase of potential users in developing countries. The proposal is applicable to any type of prosthetic-knee architecture.

The paper is organized as follows. Section 2 illustrates the proposed method. Section 3 reports the results, and Section 4 discusses them. Finally, Section 5 draws the conclusions.

## 2. Materials and Methods

**Figure 2:**
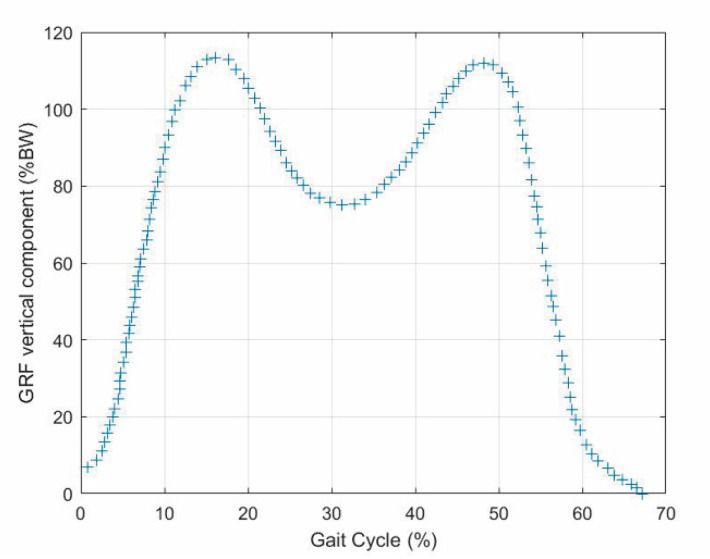
Ground reaction force (GRF) vertical component in percentage of body weight (BW) as a function of the gait-cycle percentage obtained from the dataset reported in [18].

**Figure 3:**
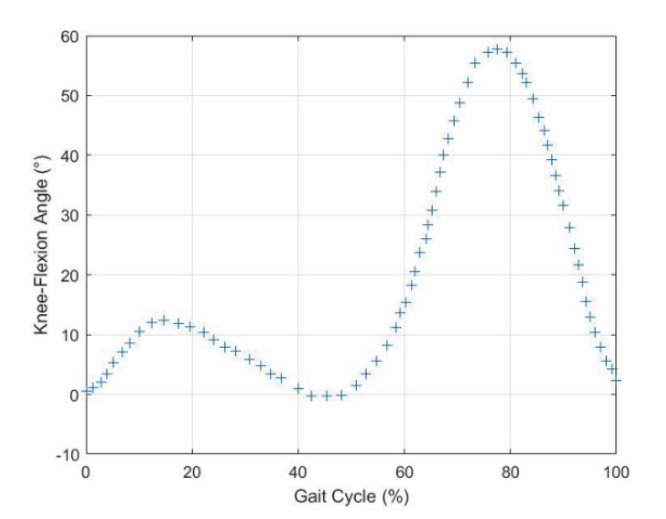
Diagram of the knee-flexion angle as a function of the gait-cycle percentage obtained from the dataset reported in [34].

**Figure 4:**
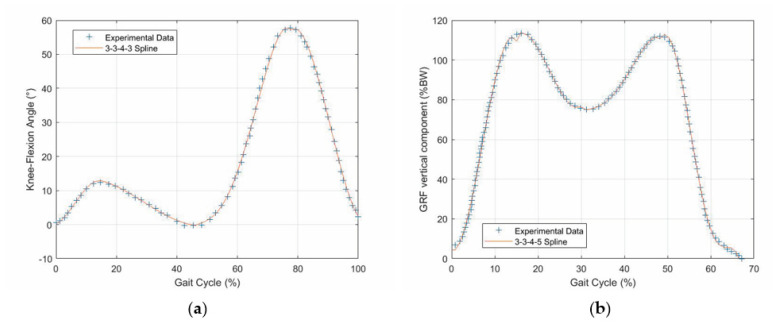
Curve fitting: (**a**) knee-flexion angle and (**b**) GRF vertical component.

**Figure 5:**
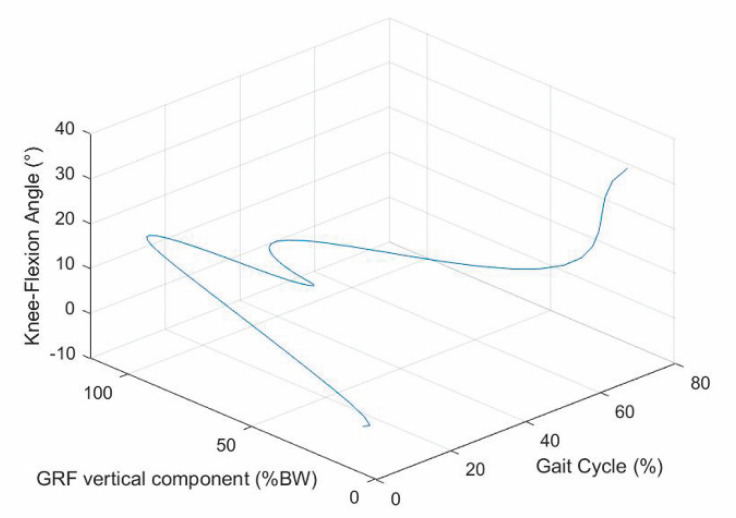
Spatial curve relating knee-flexion angle, GRF vertical component, and gait-cycle percentage.

The values reported in [18] for the GRF vertical component (Figure 2) and those reported in [34] for the knee flexion–extension (Figure 3) as functions of the gait-cycle percentage have been used to simulate datasets measured on a specific patient during normal walking. Two continuous curves, representable through algebraic equations, which give the gait parameters as a function of the gait-cycle percentage, have been generated by means of polynomial regressions on these datasets. In particular, these data have been imported into MATLAB, and a polynomial regression with the least-square method has been implemented on each monotonic part of the two data sets, thus obtaining two piecewise polynomials (splines). For the knee-flexion angle, four monotonic parts are identifiable^2^, and the best fitting was obtained with a 3-3-4-3 spline^3^ (Figure 4a). For the GRF vertical component, four monotonic parts are identifiable^4^, and the best fitting was obtained with a 3-3-4-5 spline (Figure 4b). In the stance phase, these two fitting curves, which both have the gait-cycle percentage on the abscissa, can be combined into a unique spatial curve (Figure 5) to state a one-to-one correspondence between any two variables among the knee-flexion angle, GRF vertical component, and gait-cycle percentage. Such a curve makes a possible control system able to roughly determine the knee-flexion angle and the gait-cycle percentage by measuring only the GRF vertical component. The same GRF measure, if timed, gives the gait cadence. This procedure can be directly implemented on datasets measured on a specific patient; then, the results can be used to adjust the parameters of the prosthesis control system.

### 2.1. Detection of Gait-Cycle Events

**Figure 6:**
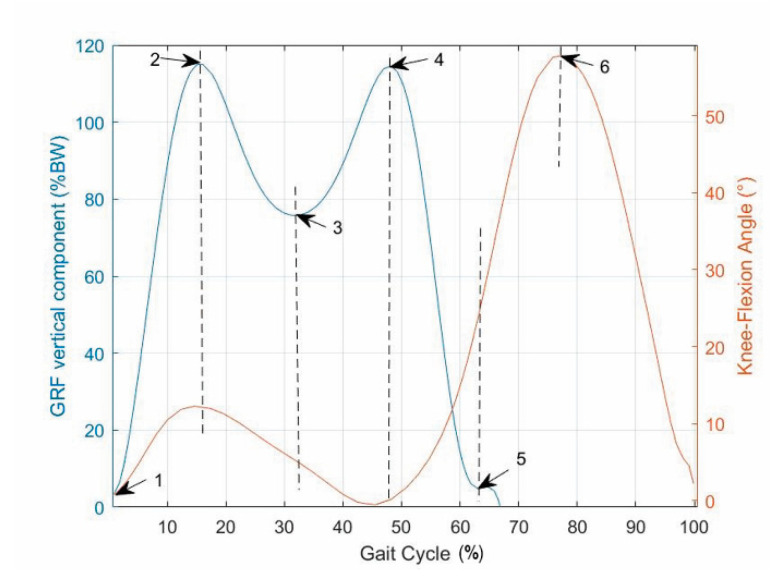
Identification of the gait-cycle events: superposition of the two fitting curves (i.e., knee-flexion angle and GRF vertical component as functions of the gait-cycle percentage).

The superposition of the two above-deduced fitting curves (Figure 6) reveals that:

- A first flexion of the knee starts at the IC event (1st event), which is easily identified by the transition of the GRF vertical component from zero to a positive value, increases with the GRF increase and reaches its maximum a bit earlier than the 1st peak of the GRF vertical component (2nd event). This is because the knee flexion somehow compensates for the shock of the sudden appearance of a non-null GRF and gives a smooth transition from the swing phase to the stance phase.
- The first knee flexion is followed by a complete knee extension that has its middle configuration at the minimum of the GRF vertical component (3rd event) and approximately terminates when the GRF vertical component reaches its 2nd GRF peak (4th event), which is at the beginning of the second double-support period.
- A second knee flexion then starts, which accompanies the decrease of the GRF vertical component until the TO event (5th event), easily identified by the transition of the GRF vertical component from a positive value to zero, and continues during the swing phase until the reaching of a maximum flexion angle that occurs nearly at the middle of the swing phase (6th event).
- The second knee flexion is followed by a second knee extension that terminates at the next IC event (1st event of the next cycle).

The above analysis highlights that six events must be detected to monitor and control the configuration of a possible prosthesis and that only the 6th cannot be detected through the GRF measure. The 6th event, which is the reaching of the maximum knee flexion during the swing phase, could be detected/controlled through an adjustable limit switch inserted in the prosthesis’ knee joint that limits the maximum knee flexion according to the cadence [34] in order to mimic the asymptomatic walking.

**Figure 7:**
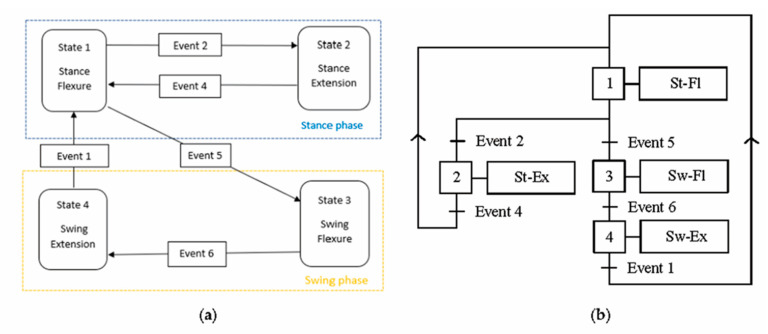
Modeling of the prosthesis control scheme: (**a**) finite-state machine and (**b**) sequential functional chart (St: stance; Sw: swing; Fl: flexure; Ex: extension).

Table 1 summarizes the reference events to use in the prosthesis control, and Figure 7 shows the control scheme for the prosthesis with a finite-state machine and a sequential functional chart.

### 2.2. Detection of the Longitudinal Slope

The datasets used in the above analysis refer to the normal gait on a flat surface. When the surface has a positive (uphill walking)/negative (downhill walking) longitudinal slope, the patterns both of the GRF vertical component and of the knee-flexion angle vary with the slope [1,35]. Consequently, the prosthesis control has to detect the slope change and, accordingly, has to change the prosthesis behavior.

The literature [1,35] shows that:

- In uphill walking, the GRF vertical component still has two peaks, but the 2nd peak is higher than the 1st, and the difference increases with the slope, whereas the knee-flexion angle still has two flexions and two extensions, but the knee flexion at the IC increases with the slope;
- In downhill walking, the GRF vertical component still has two peaks, but the 1st peak is higher than the 2nd, and the difference increases with the slope, whereas the knee-flexion angle keeps the same IC value, but all the intermediate values are amplified with an amplification factor that increases with the slope;
- Both in uphill and in downhill walking the minimum value between the two peaks does not change appreciably.

**Figure 8:**
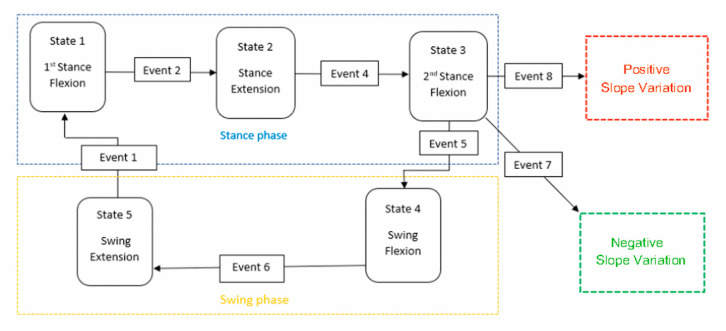
Finite-state-machine modeling of the prosthesis control scheme that takes into account the longitudinal slope of the walking.

These observations bring to choice the difference, ΔF, between the maximum GRF value at the 2nd peak and the maximum GRF value at the 1st peak as a reference variable for detecting the slope. Indeed, ΔF will be positive with a positive slope (uphill walking) and negative with a negative slope (downhill walking). Therefore, the two additional events reported in Table 2 can be added, and the finite-state machine of the prosthesis control system becomes that reported in Figure 8. With reference to Figure 8, once either of Events 7 or 8 is detected, the reference curves (knee-flexion angle, ankle-joint torque, etc.) the prosthesis control system uses to control the prosthesis behavior are changed by taking the data from a memorized database, where these curves are recorded through the coefficients of the polynomial regressions computed during the prosthesis calibration. The modified reference curves are then used after the next event 1 (IC event). Of course, all the calibration values that modify the knee-flexion fitting curves according to the slope must be adjusted on the patient during his/her rehabilitation.

### 2.3. Effects of Cadence

The measure of the GRF, if timed, provides the gait cadence. Indeed, since the time interval, Δt_1_, between two successive IC events corresponds to two steps (i.e., one stride), the cadence is 2/Δt_1_ steps/s, and the average value of the cadence on a number, n, of strides is 2n/Δt_n_, where Δt_n_ is the time interval between the first and the (n + 1)th IC events. All the digital control systems have a clock that synchronizes its actions on the controlled system; consequently, the assumption that the cadence measurement is included in the GRF measure is plausible and does not imply the addition of further sensors.

Cadence influences all the kinetic/kinematic parameters of the gait cycle [34,36,37,38]. In particular, in the GRF vertical components, the 1st peak increases, and, simultaneously, the minimum between the two peaks decreases as the cadence increases, while the 2nd peak substantially does not change; additionally, in the knee-flexion angle, the maximum flexion angle increases with the cadence increase. Eventually, the gait-cycle percentage covered by the stance phase decreases with the increase of the cadence.

Consequently, by introducing the following calibration procedure:

(i) The “normal” walking cadence of the patient is determined as the one at which the two GRF peaks are about equal;
(ii) The reference peak difference ΔF_0_, the maximum knee-flexion angle, etc. at each cadence are determined (e.g., through a regression on a finite number of measures on the patient);
(iii) The parameter ΔF that appears in Table 2 for identifying a possible slope is redefined as follows

ΔF = ΔF_0_ − ΔF_m_ (1)

where ΔF_m_ is the peak difference measured in real time during walking; the prosthesis control system can have the measured cadence, as a primary reference parameter, and the longitudinal slope, as a secondary reference parameter, for identifying which patterns for the knee-flexion angle must be used in the prosthesis control without changing the finite-state machine of Figure 8.

### 2.4. Control Algorithm

The above procedure (Figure 7 and Figure 8) for making the prosthesis control system able to identify the current configuration of the lower limb during walking is based on real-time GRF measures and on a database (memorized in the controller) containing a set of reference data/curves obtained by calibrating some prosthesis parameters through direct measurements on the patient during his/her rehabilitation training. These data bring to delineate the following control algorithm.

Step 1: The cadence is measured and, from the database, the values of ΔF_0_ to use in Equation (1) and of all the other reference parameters (e.g., the maximum knee flexion to use for adjusting knee’s limit switch) depending on the cadence are determined;

Step 2: The output data of Step 1 and the ΔF obtained from Equation (1) are used for selecting, from the database, the reference knee-flexion-angle curve to use for identifying the current knee configuration as a function of the real-time GRF measure (Figure 7 and Figure 8);

Step 3: The control system uses the measured GRF and the selected knee-flection-angle curve as input data of a simple program. This program solves the inverse dynamics of the lower limb with a prosthesis for determining the torque that the active or semiactive actuation system of the prosthesis has to apply to the prosthetic knee for making the limb mimic the selected knee-flection-angle curve.

It is worth noting that all the GRF components can be measured through load cells inserted in the prosthetic ankle of the prosthesis, which also provides the ankle-joint torque.

## 3. Results

**Figure 9:**
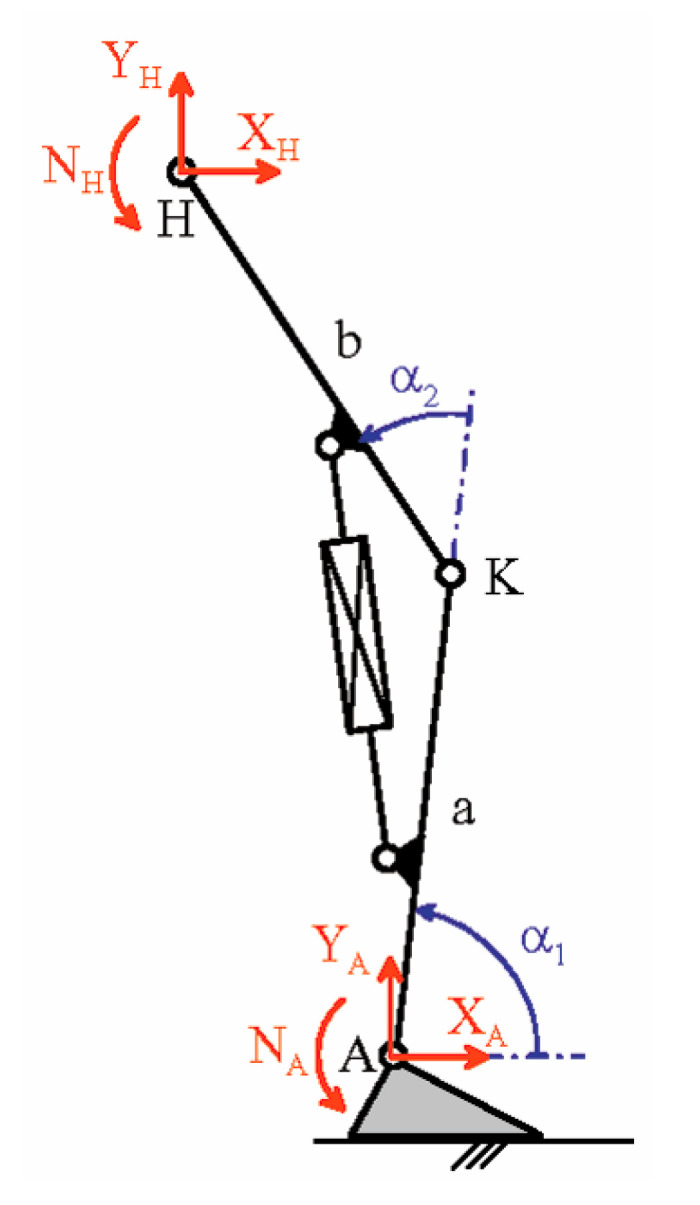
Planar model of the lower limb with a prosthesis (a = b = 0.5 m).

**Figure 10:**
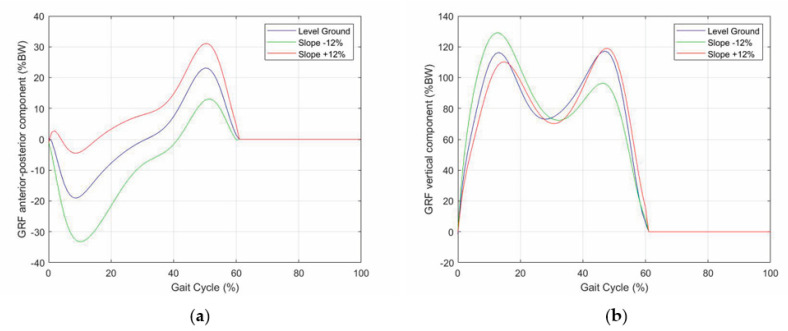
GRF components as a function of the gait-cycle percentage for three different longitudinal slopes: (**a**) anterior-posterior component, and (**b**) vertical component.

**Figure 11:**
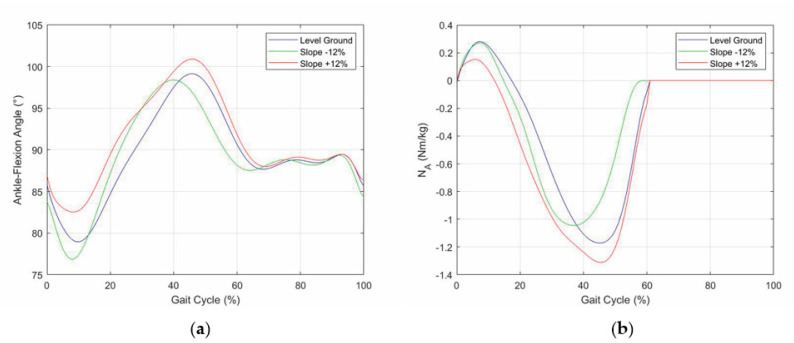
Prosthetic ankle data: (**a**) dorsi-plantar flexion angle and (**b**) ankle-joint torque.

**Figure 12:**
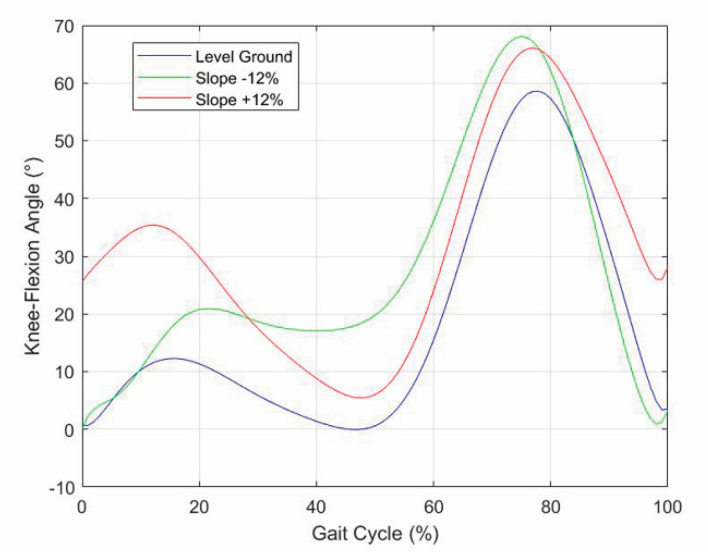
Knee-flexion angle for three different longitudinal slopes of the walkway.

This section presents the validation results of the above-defined control logic obtained through an ad-hoc simulation program developed in MATLAB, which resorts to the planar model of the lower limb with a prosthesis shown in Figure 9. In these simulations, the database measured on the patient is replaced by some published datasets of the APSIC project [1,23,39]. Figure 10, Figure 11 and Figure 12 show the continuous curves, reconstructed by means of polynomial regressions from the APSIC datasets, for level ground, slope +12%, and slope −12% of the GRF anterior–posterior and vertical components, of the prosthetic ankle [23], adopted in the model of Figure 9, and of the knee flexion, respectively.

The chosen datasets refer to walking speeds that are common in daily-life activities. These walking speeds are not high; consequently, the inertia forces are much lower than the other loads and, in the solution of the inverse dynamics problem, will be neglected. Assuming the “absence” of inertia forces in the prosthetic foot equilibrium equations makes the force system that the foot applies to the shin through the ankle equivalent to GRF; that is, the resultant force of this system is equal to the GRF, and its resultant moment, which is equal to the ankle torque, N_A_, arises from the misalignment between these two forces. So, in the model of Figure 9, point A is indeed the rotation center of the ankle, the shape of the foot does not affect the computation, and the components Y_A_ and X_A_ are the vertical and the anterior–posterior components of the GRF, respectively. The force equilibrium of the whole limb without inertia forces also reveals that the components Y_H_ and X_H_ of the force applied to the hip must be equal and opposite to Y_A_ and X_A_ (i.e., Y_H_ = −Y_A_ and X_H_ = −X_A_) and the internal torque of the hip, N_H_, is computable from the moment equilibrium equation about H of the whole limb; that is:

N_H_ + N_A_ − Y_A_[b cos(α_1_ + α_2_) + a cos(α_1_)] + X_A_[b sin(α_1_ + α_2_) + a sin(α_1_)] = 0 (2)

which gives

N_H_ = Y_A_[b cos(α_1_ + α_2_) + a cos(α_1_)] − N_A_ − X_A_[b sin(α_1_ + α_2_) + a sin(α_1_)] (3)

**Figure 13:**
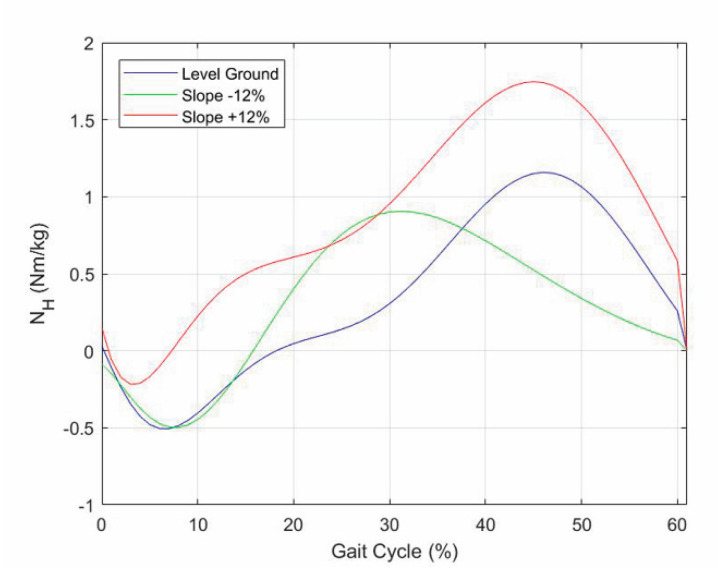
Internal torque, N_H_, of the hip that the patient must apply during the stance phase to walk with the prosthetic knee on walkways with three different longitudinal slopes.

The torque N_H_ is applied by the patient during walking. Figure 13 shows the values of N_H_ computed through Formula (3), where the values of X_A_, Y_A_, N_A_, α_1_ and α_2_ are those reported in the datasets displayed in Figure 10, Figure 11 and Figure 12 (Supplementary Data).

Eventually, the internal torque, M_K_, of the knee, which the prosthesis actuation system must provide, is computable from the moment equilibrium equation about point K of the prosthetic shank, that is:

M_K_ + N_A_ − Y_A_ a cos(α_1_) + X_A_ a sin(α_1_) = 0 (4)

which gives

M_K_ = Y_A_ a cos(α_1_) − N_A_ − X_A_ a sin(α_1_) (5)

**Figure 14:**
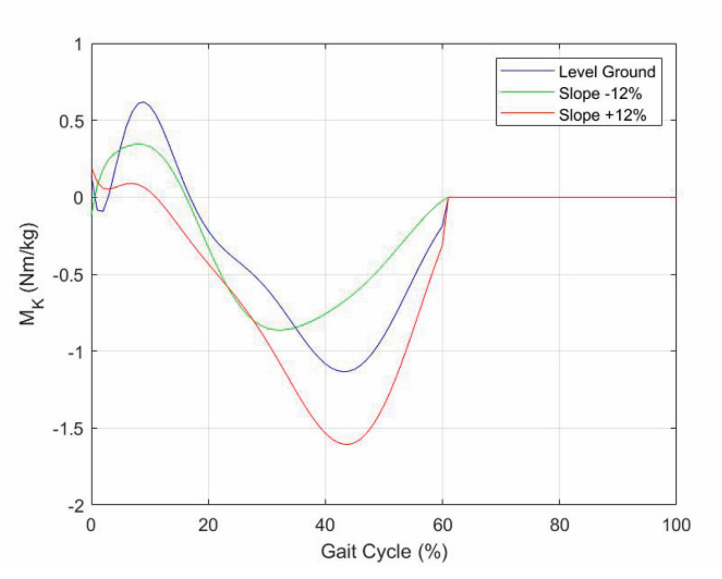
Internal torque, M_K_, of the knee that the prosthesis actuation system must provide for making the prosthetic knee mimic the asymptomatic motion on walkways with three different longitudinal slopes.

Figure 14 shows the values of M_K_ computed through Formula (5), where the values of X_A_, Y_A_, N_A_, and α _1_ are those reported in the datasets displayed in Figure 10 and Figure 11.

## 4. Discussion

The above-reported results prove that, for walking speeds of daily-life activities, it is possible to extract the pieces of information necessary to control an active or semiactive knee prosthesis by measuring only the GRF, for instance, by means of load cells inserted in the prosthetic ankle. The comparison of the internal torque, N_H_, of the hip that the patient has to apply in the case of a limb with a prosthesis (Figure 13), and in the case of a healthy limb ([19], p. 659), also reveals that they are similar. This observation allows concluding that, over mimicking the motion of a healthy limb, the proposed control procedure does not overload/stress the patient.

The proposal does not consider a particular actuation system of the active/semiactive knee prosthesis. Therefore, it can be implemented on any actuation system that is able to generate the knee-torque values, M_K_, as shown in Figure 14.

**Figure 15:**
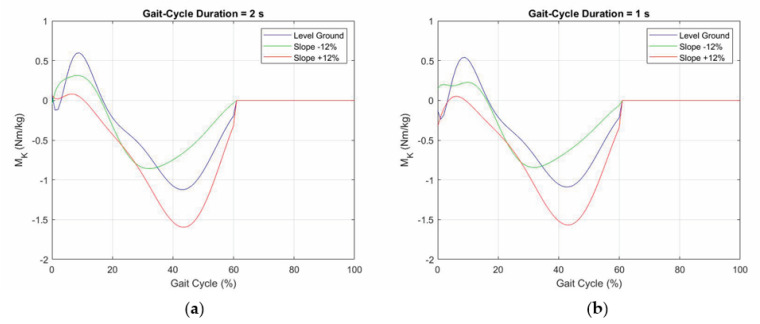
Internal torque, M_K_, of the knee prosthesis computed with Equation (6) for J = 0.274 kg m^2^ and two gait-cycle durations: (**a**) 2 s and (**b**) 1 s.

The hypothesis that the inertia forces are negligible at daily-life walking speed, which greatly simplifies the control software, needs a deeper discussion. If the inertia forces are considered, the following equation replaces Equation (5)

(6) $$M_{K}\;=\;Y_{A}\;a\;\cos(\alpha_{1})\;-\;N_{A}\;-\;X_{A}\;a\;\sin(\alpha_{1})\;+\;J\;\frac{d^{2}\alpha_{1}}{dt^{2}}$$

where J is the inertia moment of the prosthetic shank. Smith et al. [40] report realistic values of J. According to [40], J = 0.274 kg m^2^ has been chosen, and Equation (6) has been used to compute M_K_ for 2 s and 1 s of gait-cycle duration, which are durations that cover daily-life walking speeds [34]. The results of these computations are reported in Figure 15. The comparison of the diagrams reported in Figure 14 and Figure 15a,b shows that the variations are really small. Further simulations with gait-cycle durations under 1 s show that distortions are negligible until 0.75 s, which roughly corresponds to 1.92 m/s (=6.91 km/h) of walking speed and is a limit value outside of daily-life activities’ walking speeds. Consequently, the fact that inertia forces are negligible at daily-life walking speed is confirmed.

In order to evaluate if the proposed control logic is implementable in real time, the computation burden has to be determined. The only computations the controller has to perform in real time are those necessary to compute the torque M_K_ by means of Equation (5). Such computations involve 30 FLOP for computing the two trigonometric functions plus 6 FLOP for the remaining sums/subtractions/multiplications. Over the 36 FLOP for evaluating Equation (5), the preliminary computation of the ankle-flexion angle from the memorized polynomial coefficients requires a computation burden that depends on the polynomial degree and, for polynomial degrees lower than 13 (which is definitely much more than the actual degrees used in the regressions), it requires 90 more FLOP. Consequently, 126 FLOP (=90 + 36) are necessary for each M_K_ evaluation. This result allows concluding that, in a gait cycle with a duration of 0.75 s (i.e., for a walking speed faster than those of daily-life activities), 1000 computations per gait cycle would require a computation power of only 168,000 FLOP/s to implement the control algorithm in real time. An old microprocessor such as the Intel 80,486 (dated back on 1989), with a clock of 16 MHz, 0.128 FLOP/clock-cycle, and 32 bit of precision, provides 2.048 MFPLOP/s of computation power at 32 bits, which is definitely much more than 168,000 FLOP/s. Therefore, the simplicity of the algorithm guarantees its real-time implementation and the possibility of using cheap hardware components.

Eventually, the adopted validation technique deserves a final discussion. Indeed, one could object that, differently from the simulations presented above, the noise in the real-time measurements could compromise the real possibility of using the proposed control logic. On this point, it is worth stressing that, in the proposed control logic, only the GRF is measured in real time. All the other curves are fitting curves that come from a database (memorized in the controller), where these curves are recorded through the coefficients of the polynomial regressions computed during the prosthesis calibration; consequently, they are practically the same as those used in the above validation technique.

The noise in the measured GRF, which mainly comes from the shock accompanying the IC event, can also be managed through either analog or digital filters. In particular, it introduces signal distortions, the spectral components of which have frequencies much higher than those of the GRF clean signal, the main spectral components of which have frequencies lower than 25 Hz. Actually, a 0.75 s gait-cycle duration corresponds to 1.33 Hz; consequently, cutting the signal at 25 Hz corresponds to keeping the first 18 spectral components in the worst case, which is much more than necessary. For instance, the C-Leg Ottobock [33] uses a sampling rate of 50 Hz for acquiring signals, which, for the Shannon theorem, means keeping frequency components not higher than 25 Hz. One simple digital filter could be the use of the average of 10 sequentially acquired GRF values for replacing the GRF value at the end of the acquisition time. This criterion would require the acquisition of ten times more values than those that are really processed and 10 FLOP more for computing the average value. In the case of 1000 computations per gait cycle and 0.75 s of gait-cycle duration, this yields 13,333 FLOP/s to add to the above-computed 168,000 FLOP/s, which gives a total computation burden of 181,333 FLOP/s, still much lower than the computation power of an old microprocessor. Therefore, the assumption that the real-time-measured GRF signal is smooth is realistic, and its usage in the above-reported validation technique, based on simulated input data, is correct.

## 5. Conclusions

By reviewing the data reported in the literature for the gait-cycle kinematics/kinetics, a control logic has been devised for extracting the pieces of information necessary to control an active or semiactive knee prosthesis from real-time GRF measurements. Since real-time GRF measurements can be done by means of load cells inserted in the prosthetic ankle, the proposed control logic simplifies the prosthesis hardware.

In particular, the proposed control logic is able to identify the current limb configuration for different longitudinal slopes of the walkway and for different cadences, provided that the walking speed falls in the range usually spanned in daily-life activities. It then uses this information to compute the knee torque that the actuation system of the prosthesis has to apply.

The implementation of this proposal relies on datasets measured directly on the patient during his/her rehabilitation training and on general motion patterns, and it does not require a particular actuation system of the prosthesis.

The validation of the proposal has been done through simulations on a planar model. The results of these simulations showed that its implementation yields hip-joint internal torques that are comparable with those of a healthy limb, thus avoiding overload/stress for the patient.

## Figures and Tables

**Table 1:** Detection of gait-cycle events based on GRF-vertical-component measurements and the knee-flexion limit switch.

Detected Condition (t = Time Instant)	Event (Number/Name)	Gait-Cycle Percentage (%)
GRF(t − dt) = 0 & GRF(t) > 0	1/IC	0
GRF (t − dt) < GRF(t) > GRF(t + dt)	2/1st GRF peak	15
GRF(t − dt) > GRF(t) < GRF(t + dt)	3/heel rise	32
GRF(t − dt) < GRF(t) > GRF(t + dt)	4/2nd GRF peak	47
GRF(t) > 0 & GRF(t + dt) = 0	5/TO	64
Limit Switch Reached	6/max knee flexion	78

**Table 2:** Detection of the longitudinal slope based on GRF-vertical-component measurements.

Detected Condition (i = Cycle Index)	Event (Number)	Slope Variation (Sign)
ΔF_i_ > ΔF_i+1_	7	Negative
ΔF_i+1_ > ΔF_i_	8	Positive

## Data Availability

The data sets used in this work have been uploaded as “Supplementary Materials” accompanying the paper.

## Footnotes

1 According to ([19], p. 632), the reference data for “normal” walking are 1.37 m/s for the walking speed, 1.87 steps/s (110 steps/min) of step rate (cadence), and 0.72 m of step length.

2 The first between the IC event and the 1st peak occurrence, the second between the 1st peak occurrence and the central minimum, the third between the central minimum and the 2nd peak occurrence, and the fourth between the 2nd peak occurrence and the next IC event.

3 The numbers separated by the hyphens indicate the degrees of the polynomials that compose the spline.

4 The first between the IC event and the 1st peak occurrence, the second between the 1st peak occurrence and the central minimum, the third between the central minimum and the 2nd peak occurrence, and the fourth between the 2nd peak occurrence and the TO event.

## Supplementary Materials

The files containing the datasets used in this paper and the MATLAB program used to generate Figure 10, Figure 11, Figure 12, Figure 13, Figure 14 and Figure 15 are in the zipped file MatLabProgram+Data.zip available online at https://www.mdpi.com/article/10.3390/biomimetics6020022/s1.
